# Effects of Branched-Chain Fatty Acids Derived from Yak Ghee on Lipid Metabolism and the Gut Microbiota in Normal-Fat Diet-Fed Mice

**DOI:** 10.3390/molecules28207222

**Published:** 2023-10-23

**Authors:** Ting Tan, Yihao Luo, Wancheng Sun, Xiaoxiao Li

**Affiliations:** Animal Science Department, College of Agriculture and Animal Husbandry, Qinghai University, Ning Da Road 251, Xining 810016, China; tanting0618@outlook.com (T.T.); 1997990013@qhu.edu.cn (W.S.); lixiaoxiao0618@outlook.com (X.L.)

**Keywords:** yak ghee, branched chain fatty acids (BCFAs), gene expression, fat metabolism, 16S rDNA, KEGG metabolic pathway

## Abstract

Branched-chain fatty acids (BCFAs) are natural components with a variety of biological activities. However, the regulation of lipid metabolism by BCFAs is unknown. It was dedicated to examining the impacts of BCFAs inferred from yak ghee on the expression of qualities related to lipid metabolism, natural pathways, and intestinal microbiota in mice. The treatment group (purified BCFAs from yak ghee) exhibited a decrease in cholesterol levels; a decrease in *HMGCR* levels; downregulation of *FADS1*, *FADS2*, *ACC-α*, *FAS*, *GAPT4*, *GPAM*, *ACSL1*, *THRSP*, *A-FABP*, and *PPARα* gene expression; and upregulation of *SCD1*, *ACSS1*, *FABP1*, *CPT1*, and *DGAT-1* gene expression. Gut microbiota 16S rDNA sequencing analysis showed that the treatment group improved the gut microbiota by increasing the relative abundances and increasing the short-chain fatty acid levels produced by the genera *Akkermansia*, *Clostridium*, *Lachnospiraceae*, *Lactobacillus*, *Anaerotaenia*, and *Prevotella*. After adding BCFAs to cultured breast cancer cells, pathways that were downregulated were found to be related to fatty acid degradation and fatty acid metabolism, while 20 other pathways were upregulated. Our results suggest that BCFAs reduce body fat in mice by modulating intestinal flora and lipid metabolism and modulating fatty acid metabolism in breast cancer cells.

## 1. Introduction

Yak (Bos grunniens) ghee is a dairy product from traditional yak milk and is usually made by hand with special bulk. Yak ghee is mainly consumed in the diet in milk tea and in zanba made from Qingke wheat in Tibet and Qinghai. Yak ghee is high in calories and rich in functional fatty acids, which is of great importance to herders living in the cold regions of the plateau where vegetables are scarce. Sun et al. [1] determined the composition of yak milk fat by gas chromatography, where most of it was saturated fatty acids (60.56%), and the monounsaturated fatty acid content was 35.05%, of which the contents of polyunsaturated fatty acids and BCFAs [2] were higher than those of ordinary milk. Zheng et al. found that the body weight and abdominal fat of mice with yak ghee added to the diet increased significantly, the level of triglyceride (TG) and total cholesterol (TC) in the blood increased significantly, and the mechanism was perhaps connected to the composition of fatty acids [3]. Jian found that the intake of hydrogenated soybean oil and yak ghee can significantly increase the contents of TC and TG in the liver of mice and speculated that the increase in fat anabolism in the yak ghee group may be related to the high content of saturated fatty acids in mice [4]. BCFAs are a class of fatty acids with one or more branches (mainly methyl groups) on the carbon chain, which are mainly saturated fatty acids. Current studies on BCFAs have focused on the content, identification, and biological activity of different raw materials. The BCFA content varies among samples. Xue et al. studied BCFAs and odd-chain fatty acids (OCFAs) in mammalian milk, dairy products, and vegetable oils and identified and quantified 72 fatty acids, including 16 BCFAs and 10 OCFAs, using GC/TOF-MS [5]. Ma determined the content of BCFAs in milk samples from dairy cows, buffaloes, yaks, Juanshan cattle, goats, camels, and horses by GC–MS [2]. The results showed that the total content of BCFAs in yak milk was the highest. The BCFAs in yak ghee are mainly derived from the de novo fatty acids synthesized by rumen microorganisms. Isobutyric acid, isovaleric acid, and 2-methylbutyric acid are used by rumen microorganisms to synthesize BCFAs [6]. When rumen microbes die and enter the small intestine, these fatty acids are digested, absorbed by the host, and deposited in meat and milk. Fewer BCFAs are de novo synthesized naturally from the diet or in the ruminant’s own tissues [7].

BCFAs have various biological activities, such as anti-inflammatory [8] and anticancer [9] activities. Current research indicates that the function of BCFAs is primarily anticancer. According to Yang et al. [10], iso-15:0 obtained from aged soybeans can halt the development of a number of cancer cells both in vitro and in vivo. BCFAs inhibit the synthesis of fatty acids in breast cancer cells and disrupt the integrity of SKBR-3 mitochondria in breast cancer cells, thereby inducing apoptosis [11]. Yu et al. [12] found that BCFAs can indirectly reduce edema and inflammation, and their mechanism of action is to inhibit the function of platelets and white blood cells. BCFAs have been reported to be beneficial for intestinal health in humans and monogastric animals [13,14]. According to relevant studies, BCFAs have also been shown to prevent and treatment necrotizing enterocolitis (NEC). Ran-Ressler found that BCFAs relieve NEC incidence and alter the microbiota composition [13]. Li found that the BCFAs iso-15:0 and iso-18:0 have significant lipid-lowering effects in vitro, and BCFAs can alleviate the increase in triglyceride content caused by free fatty acids [15]. Zhou et al. found that toxic substances in excessive frying oil can disrupt the body’s fatty acid anabolism by affecting the normal synthesis and metabolism of fatty acids [16]. Body weight and abdominal lipid content were significantly increased in mice fed yak ghee, while iso-15:0 and iso-18:0 significantly reduced triglyceride levels in mice in vitro. There are no studies on the effects of yak ghee BCFAs on lipid anabolism and catabolism in the body, and their regulatory mechanisms on lipid metabolism-related genes in vivo need to be studied in depth.

There are two types of fat metabolism genes related to anabolism and catabolism, which together determine the deposition of fat in animals. (1) Genes related to fatty acid synthesis are mainly fatty acid synthase (*FAS*), fatty acid desaturase 1 (*FADS1*), fatty acid desaturase 2 (*FADS2*), acetyl-CoA carboxylase α (*ACC-α*), stearyl desaturase 1 (*SCD1*), and acetyl-CoA synthetase 1 (*ACS1*). *FAS* plays a central role in lipid biosynthesis, catalyzing the amalgamation of fatty acids from acetyl-CoA, malonyl-CoA, and nicotinamide adenine dinucleotide phosphate (*NADPH*) [17]. Its overexpression can lead to the deposition of body fat, which can cause obesity [18]. *FADS1* and *FADS2* are imperative metabolic rate-limiting proteins within the amalgamation of polyunsaturated fatty acids [19]. *ACC-α* is the first rate-limiting enzyme in the fatty acid biosynthesis pathway and plays a key role in the biosynthesis of fatty acids [20]. *SCD1* is a rate-limiting enzyme that catalyzes the desaturation of saturated fatty acyl groups and the formation of double bonds at the Δ9 position of fatty acids to produce monounsaturated fatty acids [21]. *ACS1* is a mitochondrial protein included in the blend of acetyl-CoA amid the tricarboxylic corrosive cycle. Acetyl-CoA may be a precursor of fatty acid and cholesterol synthesis [22]. (2) The genes involved in the synthesis of triacylglycerol are mainly long-chain fatty acyl-CoA synthetase 1 (*ACSL1*), glycerol-3-phosphate acyltransferase 4 (*GPAT4*), diacylglycerol acyltransferase 1 (*DGAT1*), recombinant glycerol-3-phosphate acyltransferase, mitochondrial (*GPAM*), and thyroid hormone response protein (*THRSP*). Related reports have indicated that ACSL1 is an enzyme needed for the activation of fatty acid acyl-CoA by fatty acid activation [23]. Both the *GPAM* and *GPAT4* genes are part of the *GPAT* family, and they catalyze the reaction of glycerol-3-phosphate and fatty acyl-CoA. *GPATs* are key enzymes in the first step of triglyceride synthesis. *GPAM* is localized to mitochondria, and *GPAT4* is localized to the endoplasmic reticulum. *GPATs* and *DGAT* are key enzymes in the phosphoglycerol pathway, which is the major pathway for the synthesis of triglycerides in most cells [24]. *THRSP* could be a transcriptional controller included in thyroid hormone and carbohydrate-induced adipogenesis by directing the expression of rate-limiting proteins in lipid unions [25]. (3) The gene involved in cholesterol synthesis is mainly recombinant 3-hydroxy-3-methylglutaryl coenzyme A reductase (*HMGCR*). *HMGCR* is a rate-limiting enzyme for cholesterol synthesis that regulates the synthesis of cholesterol. (4) Genes related to lipid catabolism mainly include adipocyte-type fatty acid binding protein (*AFABP*) and carnitine palmitoyltransferase 1 (*CPT1*), of which *CPT1* is the rate-limiting enzyme for fatty acid β-oxidation [26]. *AFABP* is mainly expressed in adipocytes and macrophages. It takes part in the digestion system of immersed and unsaturated long-chain fatty acids and regulates the oxidation of fatty acids and the metabolism of phospholipids and triglycerides [27]. Peroxisome proliferator-activated receptor alpha (*PPARα*) could be a nuclear receptor protein that is primarily involved in translation. PPARα plays an important role in the metabolism of sugars, lipids, and proteins [28]. Its main function is to control the intake and activation of fatty acids [29]. Hepatic fatty acid-binding protein 1 (*FABP1*) is the only fatty acid-binding protein expressed in the liver and is closely related to lipid absorption in the small intestine, lipid transport in the liver, and lipoprotein metabolism. Studies have shown that *FABP1* is mainly regulated by *PPARα*. Regarding its regulation, *PPAR* stimulates fatty acid catabolism by inducing mitochondrial and peroxisome oxidation levels and *FABP1* gene expression [30]. *FABP1* also affects triglyceride synthesis and fat oxidation in the liver [31]. Studies have shown that the expression of *FABP1* greatly enhances the oxidation of BCFAs, which is stronger than absorption or esterification [32].

Currently, most of the research on yak ghee focuses on its composition and overall function, but there is little research on the specific functions of certain components in yak ghee. Yak ghee contains a variety of functional dietary fatty acids (BCFAs). Alterations in fatty acid metabolism can affect the distribution and function of membrane proteins and the level of gene expression, and the structure of fatty acids in the cellular environment can affect the fluidity of cell membranes and can alter cellular integrity and the function of cell membranes. In a previous study, the group found that BCFAs could inhibit the proliferation of obesity-related breast cancer cells but did not explore the expression of related lipid metabolism genes. In the present study, the effects of BCFAs in yak ghee on lipid metabolism were initially investigated through in vivo mouse experiments and in vitro cellular experiments to lay a theoretical foundation for subsequent experiments on obesity inhibition in mice.

The purpose of this study was, first, to add 3% BCFAs (total purity of BCFAs: 16.3%) and 10% yak ghee (total purity of BCFAs: 3.3%) to the diets of mice and to conduct a comparative study of hepatic lipid metabolism in mice with the two dietary interventions. Second, free fatty acids from yak ghee were used as a blank control group (total purity of BCFAs: 3.3%), BCFAs (total purity of BCFAs: 16.3%) were used as an experimental group, which were added into human breast cancer SK-BR-3 cells, and the expression of genes related to the lipid metabolism of breast cancer cells by BCFAs in yak ghee was analyzed by transcriptomics, which further validated the mouse liver lipid metabolism profile.

## 2. Results

### 2.1. Effect of Purified BCFAs from Yak Ghee on Body Weight and Food Intake

After feeding for four weeks, the body weight difference among the groups was not significant (*p* > 0.05), as shown in Table 1. As shown in Table 1, the total food intake of mice in the treatment group was reduced compared with the control and ghee groups, and the food utilization rate of mice in the ghee group and the treatment group was higher than that of the control group (*p* > 0.05), which indicated that the BCFAs were capable of suppressing the appetite of mice.

### 2.2. Effect of Purified BCFAs from Yak Ghee on Serum Biochemical Indices in Mice

Blood was taken from the eyeballs of mice after dissection, and the supernatant was obtained by centrifugation to determine total cholesterol (TC), triglyceride (TG), LDL cholesterol (LDL-C), and HDL cholesterol (HDL-C) levels using a kit, as shown in Table 2.

As shown in Table 2, the TC, TG, and LDL-C levels in the treatment group were lower than those in the control group, and the difference was statistically significant (*p* < 0.01), while HDL-C was significantly higher than that in the control group (*p* < 0.01). The difference between TC, TG, and LDL-C in the ghee group was higher than that in the control group, and the difference was statistically significant (*p* < 0.01), while HDL-C was lower than that in the control group (*p* < 0.01). The differences in TC, TG, LDL-C, and HDL-C between the treatment and ghee groups were statistically significant (*p* < 0.01). The results indicated that BCFAs from yak ghee could reduce TC, TG, and LDL-C, increase HDL-C, and improve lipid levels in C57BL/6 mice.

### 2.3. Profile of Gene Expression of Fatty Acid Synthesis-Related Genes

The *FAS*, *FADS1*, *FADS2*, and *ACC-α* genes were downregulated, except for the *SCD1* and *ACSS1* genes, which were upregulated in the ghee and treatment groups.

**Figure 1:**
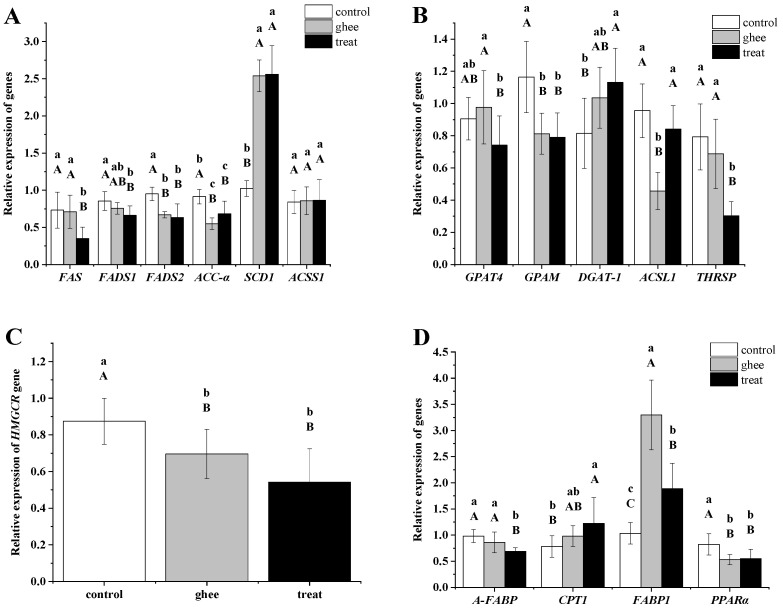
Effect of yak ghee BCFAs on gene expression in the liver of mice. Expression of genes related to fatty acid synthesis (**A**). Expression of triacylglycerol synthesis-related genes (**B**). Expression of the cholesterol synthesis-related gene *HMGCR* (**C**). Expression of genes related to lipid metabolism (**D**). Note: *FAS*, fatty acid synthase; *FADS1*, fatty acid desaturase 1; *FADS2*, fatty acid desaturase 2; *ACC-α*, acetyl-CoA carboxylase α; *SCD1*, stearyl desaturase 1; *ACSS1*, aacetyl-CoA synthetase 1; *NADPH*, nicotinamide adenine dinucleotide phosphate; *ACSL1*, acyl-CoA synthetase 1; *GPAT4*, glycerol-3-phosphate acyltransferase 4; *DGAT1*, diacylglycerol acyltransferase 1; *GPAM*, recombinant Glycerol-3-Phosphate Acyltransferase, Mitochondrial; *THRSP*, thyroid hormone response protein; *HMGCR*, recombinant 3-Hydroxy-3-Methylglutaryl Coenzyme A Reductase; *A-FABP*, adipocyte-type fatty acid binding protein; *CPT1*, carnitine palmitoyltransferase 1; *PPARα*, Peroxisome proliferator-activated receptor alpha; *FABP1*, fatty acid-binding protein 1. Lowercase letters indicate significance at the 0.05 level, and uppercase letters indicate significance at the 0.01 level. Different letters indicate significant or highly significant differences between groups.

As shown in Figure 1A, this result indicated that the expression of the *FAS*, *ACC-α*, *FADS1*, and *FADS2* genes was inhibited in the treatment group, suggesting that purifying BCFAs from yak ghee could inhibit the synthesis of fatty acids and polyunsaturated fatty acids.

The relative expression level of the *ACSS1* gene in each group was not significantly different but was mildly increased in the ghee and treatment groups (*p* > 0.05).

The *ACSS1* gene is involved in the synthesis of acetyl-CoA in the Krebs cycle [22]. The results indicate that the synthesis of acetyl-CoA can provide the substrate for the synthesis of monounsaturated fatty acids, with a significant upregulation of *SCD* gene expression.

### 2.4. Gene Expression of Genes Involved in Triacylglycerol Synthesis

In Figure 1B, the fluorescence quantification of genes showed that the expression levels of *GPAT4*, *GPAM*, and *THRSP* genes in the BCFAs group were much lower than those in the ghee and control groups (*p* < 0.01). The expression level of the *ACSL1* gene was lower than that of the control group and higher than that of the ghee group. The expression level of the *DGAT-1* gene was higher than that of the control and ghee groups in both groups.

Both the *GPAM* and the *GPAT4* genes are *GPATs*, and they are key enzymes in the first step of triglyceride synthesis. *GPAM* is localized to mitochondria, and *GPAT4* is localized to the endoplasmic reticulum [24]. *GPATs* and *DGAT* are key enzymes in the glycerol phosphate pathway, indicating that the blood lipid content was reduced by inhibiting the first step of the synthesis of triglycerides by the glycerol phosphate pathway with supplementation with purified BCFAs from yak ghee in the diet of mice.

*ACSL1* is related to the activation of the long-chain fatty acid acyl-CoA [23]. The relative expression of *ACSL1* in the ghee group was lower than that of the other two groups, indicating that purified BCFAs from yak ghee can be used in the mouse diet to inhibit the production of long-chain fatty acyl-CoA in the body.

The *THRSP* gene is involved in thyroid hormone and carbohydrate-induced lipogenesis [25]. Studies have shown that the expression level of *FAS* increases with increasing *THRSP* expression [33]. The relative expression of the gene in the treatment group was lower than that in the other two groups, indicating that the hypolipidemic effect of the treatment group was related to the downregulation of *THRSP*. This result suggested that the hypolipidemic effect of purified BCFAs may occur by reducing the synthesis of fat induced by carbohydrates and thyroid hormones.

### 2.5. Expression of the Cholesterol Synthesis Gene HMGCR

In Figure 1C, the difference in the expression of the *HMGCR* gene between the control group and the treatment group was extremely significant (*p* < 0.01).

*HMGCR* is the rate-limiting enzyme in cholesterol biosynthesis. From the results, the expression of this gene in the treatment group was lower than that in the control group, indicating that the decrease in serum cholesterol levels in the treatment group was related to the downregulation of *HMGCR* gene expression.

### 2.6. Gene Expression of Lipid Metabolism-Related Genes

As shown in Figure 1D, according to the relative expression of the *A-FABP*, *CPT1*, *FABP1*, and *PPARα* genes, the *A-FABP* and *PPARα* genes were significantly down-regulated in the BCFAs group compared with the control group (*p* < 0.01), while the *CPT1* and *FABP1* genes were up-regulated (*p* < 0.01).

The *A-FABP* gene was mainly expressed in adipocytes. From the results, we speculated that the adipocytes in the liver of the treatment group had relatively low lipid synthesis. In Li’s study, nonalcoholic fatty liver was associated with fat accumulation in the liver, while BCFAs played a role in alleviating triglyceride accumulation [15]. It was confirmed that BCFAs alleviate triglyceride accumulation.

The difference in the expression of the *CPT1* gene between the control group and the treatment group was extremely significant (*p* < 0.01). The CPT1 gene is the rate-limiting enzyme in the β-oxidation of fatty acids [26]. The expression of the gene in the treatment group was significantly higher than that in the remaining two groups, indicating that supplementation of the diet of mice with BCFAs purified from yak ghee can increase the β-oxidation of fatty acids, thereby lowering blood lipids.

*FABP1* is involved in lipid transport in the liver, and the gene expression of *FABP1* greatly enhances the oxidation of the pathway and fatty acid esterification and is mainly regulated by *PPARα* promoter transcription initiation [31]. From the results, the expression of the *FABP1* gene in the treatment group and the ghee group was significantly higher than that in the control group, indicating that purified BCFAs in the diet of mice can improve lipid transport and metabolism in the liver, which, due to the increase in BCFA content, can enhance the expression of *FABP1*.

**Figure 2:**
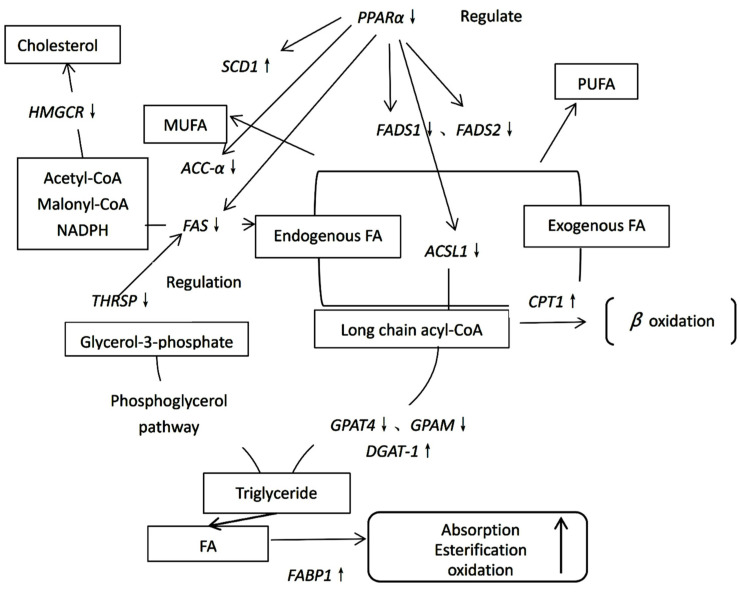
Schematic diagram of the effect of purified BCFAs from yak ghee on the fatty acid metabolism pathways in mice.

*PPARα* may be related to transcription factors that regulate mitochondrial biogenesis and energy metabolism. The upregulation of *PPARα* in the midst of lactation concurs with the point-by-point increases in the numbers and turnover rate of mitochondria in lactating mammary tissue [34]; that is, downregulation of *PPARα* gene expression may reduce lipid synthesis. From the results, the downregulation of *PPARα* may result in a decrease in lipid synthesis in the treatment group fed purified BCFAs in the diet. In short, the network of the effect of purified BCFAs from yak ghee on the fatty acid metabolism pathway of mice is shown in Figure 2.

### 2.7. Analysis of the Diversity of Gut Microbiota in Each Group of Mice

Alpha diversity is commonly used to measure species richness in community ecology and is a comprehensive indicator of species richness and uniformity. Differences in the diversity of the mouse gut flora were analyzed by the Chao1, observed species, Shannon, and Simpson indices. The results showed that the differences in the Chao1 and Shannon indices between the control group and the treatment group were also significant (*p* < 0.05), as shown in Table 3. The Chao1 index and Shannon index were lower in the ghee and treatment groups than in the control group, suggesting a decrease in the number of species in the intestinal flora and lower community diversity in mice after ghee and BCFA interventions.

**Figure 3:**
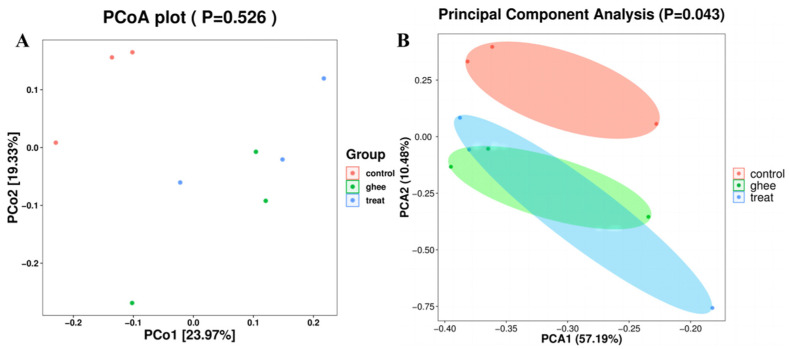
Beta-diversity analysis of mouse intestinal flora structure. PCoA analysis of mouse gut flora structure (**A**). PCA of mouse gut flora structure (**B**).

Beta diversity mainly utilizes the species composition and abundance information of each sample to reflect the relationship between the samples. Principal coordinate analysis (PCoA) and principal component analysis (PCA) were used to characterize the differences in the structure of the intestinal flora among the three groups of mice. The PCoA and PCA of the gut flora of mice were analyzed. In Figure 3A,B, the distribution of the control group was significantly different from that of the ghee group and the treatment group (*p* < 0.05).

### 2.8. Analysis of the Species Composition of the Intestinal Flora of Mice

#### 2.8.1. Relative Abundance of Major Phyla of the Intestinal Flora

**Figure 4:**
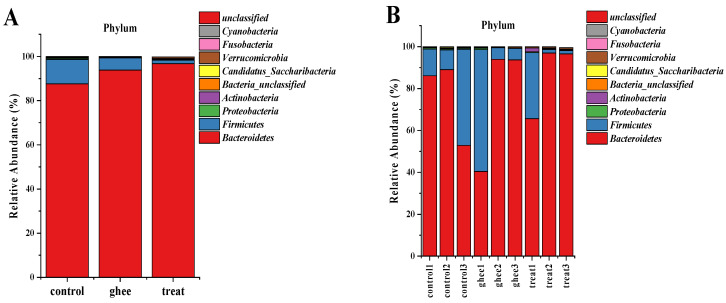

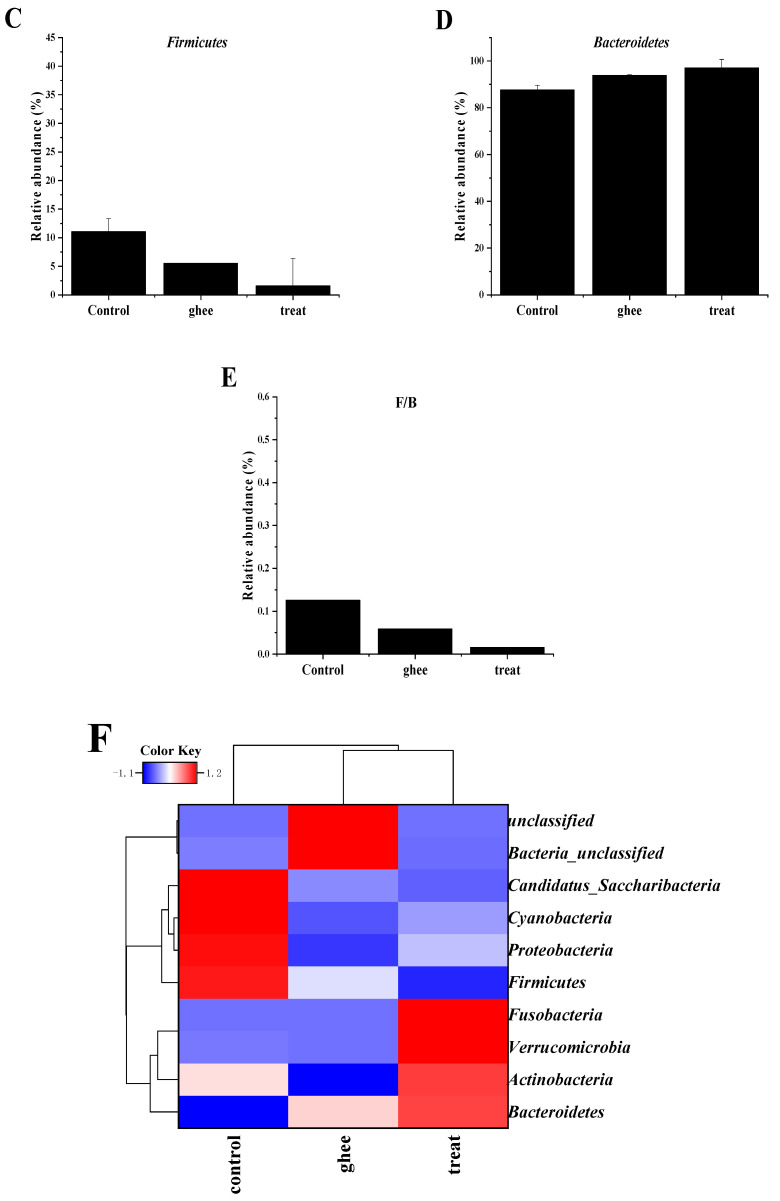
Effect of ghee and BCFAs intervention on the change in relative abundance of intestinal bacterial flora gate levels in mice. Relative abundance at the phylum level (**A**,**B**). Relative abundance of *Firmicutes* (**C**). Relative abundance of *Bacteroidetes* (**D**). F/B values (**E**). Clustering heatmap at the phylum level (**F**).

At the phylum level, the intestinal flora consisted of *Bacteroidetes*, *Firmicutes*, *Proteobacteria*, *Actinobacteria*, *Candidatus_Saccharibacteria*, *Verrucomicrobia*, etc., as shown in Figure 4A,B. As shown in Table 4, in the control group, *Bacteroidetes* and *Firmicutes* were the major dominant phyla, with relative abundances of 87.601% and 11.052%, respectively. The relative abundance of the Ascomycetes phylum in the ghee and treatment groups was lower than that of the control group, and the difference was significant. *Verrucomicrobia* in the treatment group showed significant proliferation compared with the control and ghee groups. As shown in the clustered heatmaps in Figure 4C–F, the relative abundance of *Bacteroidetes* increased and the relative abundance of *Firmicutes* decreased in the ghee and treatment groups compared with the control group. F/B can reflect the extent to which the flora helps in the absorption of heat in the body. A decreased F/B ratio in the ghee and treatment groups helps the body burn energy and has a positive effect on the treatment of obesity.

#### 2.8.2. Relative Abundance of Major Genera and Species of Intestinal Flora

**Figure 5:**
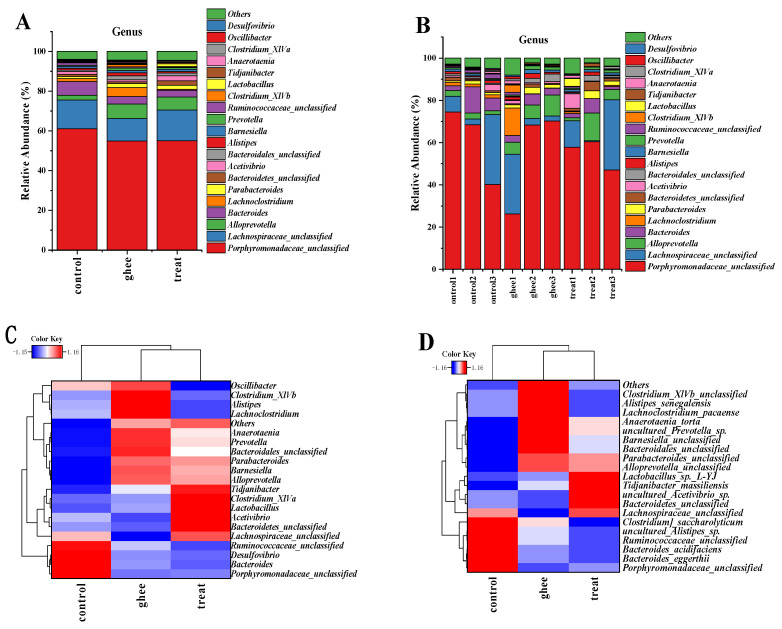
Effect of ghee and BCFAs intervention on changes in relative abundance of intestinal flora genus levels in mice. Relative abundance at the genus level (**A**,**B**). Clustering heatmap at the genus level (**C**). Clustering heatmap at the species level (**D**).

As Figure 5A,B show only the top 20 genera with high relative abundance, at the genus level, the gut flora consists of *Porphyromonadaceae_unclassified*, *Lachnospiraceae_unclassified*, *Alloprevotella*, *Bacteroides*, *Lachnoclostridium*, *Parabacteroides*, *Bacteroidetes_unclassified*, *Acetivibrio*, *Bacteroidales_unclassified*, *Alistipes*, *Barnesiella*, *Prevotella*, *Ruminococcaceae_unclassified*, *Clostridium_XlVb*, *Lactobacillus*, *Tidjanibacter*, *Anaerotaenia*, *Clostridium_XlVa*, *Oscillibacter*, *Desulfovibrio*, and 111 other genera. In Table S4, the relative abundances of the intestinal flora exhibited more genera that were increased and fewer genera that were decreased in the treatment and ghee groups than in the control group.

The results showed that the relative abundance of *Helicobacter* in the treatment group and the ghee group was lower than that in the control group, and the difference was significant. The relative abundance of *Bilophila* in the treatment group was higher than that in the control group and the ghee group, and the difference was significant. The relative abundance of *Akkermansia* in the treatment group was significantly higher than that in the control group and the ghee group. *Lactobacillus* in the treatment group and the ghee group showed significant differences compared with the control group. *Lactobacillus sp. L-YJ* and *Lactobacillus vaginalis* among the *Lactobacillus* genera were found to have significant differences in the gut flora of the three groups of mice, as shown in Table S5 and Figure 5C,D.

There were significant differences in the gut flora at the genus level among the three groups of mice, such as *Helicobacter*, *Bilophila*, *Akkermansia*, and *Lactobacillus*, as shown in Table 5.

### 2.9. Analysis of KEGG Pathways in the SK-BR-3 Transcriptome of Breast Cancer Cells

**Figure 6:**
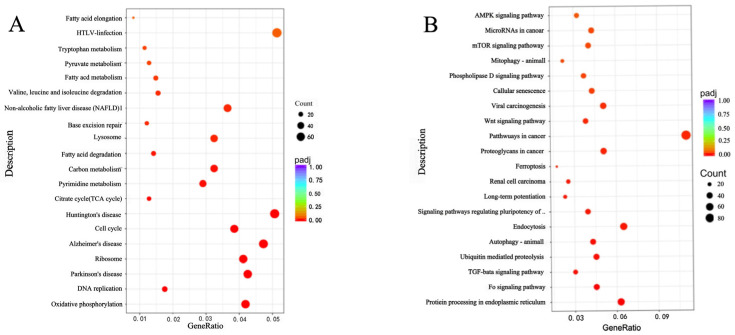
KEGG enrichment analysis bubble diagram. Metabolic pathways are downregulated in the KEGG enrichment bubble diagram (**A**). Metabolic pathways are upregulated in the KEGG enrichment bubble diagram (**B**).

The results are shown in Figure 6. Among the 20 pathways with the most significant difference, the number of genes in the enrichment analysis was close to 60 for six pathways and 20 genes for nine pathways. Pathways with fewer than 20 genes enriched were mainly associated with DNA replication, fatty acid elongation metabolism, fatty acid metabolism (synthesis, transport of fatty acids), and fatty acid degradation. The pathways with almost 60 genes enriched were mainly associated with HTLV infection, ribosomes, and oxidative phosphorylation. Pathways with between 20 and 40 enriched genes were mainly located in the cell lysosome, carbon metabolism, and pyrimidine metabolism, as shown in Table S6 and Figure 6A.

Regarding the results of the upregulated pathways by KEGG enrichment analysis in Figure 6B, the results showed that among the 20 most significant pathways with 80 genes enriched, the pathways were mainly the regulation of metabolism in cancer, cell autophagy, viral carcinogenesis, endocytosis, ferroptosis, and protein processes in the endoplasmic reticulum, as detailed in Table S7 and Figure 6B.

## 3. Discussion

According to the changes in body weight and cholesterol content among blood lipids before and after feeding, the purified BCFAs from yak ghee can significantly reduce the total cholesterol content in mice. From the analysis results of the *HMGCR* gene, it can be seen that the mouse diet can lead to a significant reduction in the expression of the *HMGCR* gene, and it is confirmed at the genetic level that supplementation of the mouse diet with purified BCFAs from yak ghee can inhibit the synthesis of cholesterol in mice. It is suggested that long-term consumption of purified BCFAs from yak ghee may prevent cardiovascular and cerebrovascular diseases.

Analysis of the *FADS1*, *FADS2*, *ACC-α*, *FAS*, and *SCD1* genes demonstrated that diets containing yak ghee and purified BCFAs from yak ghee inhibited the synthesis of polyunsaturated fatty acids in mice but improved the synthesis of monounsaturated fatty acids, ultimately reducing the synthesis of fatty acids in the bodies of mice. Analysis of the *ACSS1* gene showed that the dietary differences in mice had no effect on the amount of synthesis of acetyl-CoA in the tricarboxylic acid cycle. The *ACSL1*, *GPAT4*, *GPAM*, and *DGAT-1* gene expression results indicated that purified BCFAs from yak ghee can be used in the diet of mice to reduce body fat by inhibiting the synthesis of triglycerides in the glycerol phosphate pathway. According to the analysis of the expression of the *THRSP* gene in mouse liver, it is suggested that purified BCFAs from yak ghee can be used in the diet of mice to reduce body fat synthesis by reducing the synthesis of fat induced by thyroid hormone and carbohydrates. In general, supplementation of the mouse diet with purified BCFAs from yak ghee can reduce body fat synthesis in mice by inhibiting the glycerol pathway and reducing the thyroid hormone and carbohydrate-induced synthesis of fat. The *A-FABP* gene is mainly expressed in adipocytes. Analysis of the *A-FABP* and *CPT1* genes demonstrated that the use of purified BCFAs from yak ghee as food for mice can improve the β-oxidation of fatty acids, thus reducing the synthesis and accumulation of fat in mice. It has been confirmed from other angles that BCFAs alleviate triglyceride accumulation. According to the results of the *FABP1* gene expression analysis, yak ghee and purified BCFAs from yak ghee used in the diets of mice may promote the expression of *FABP1* and increase lipid transport and metabolism in the liver due to the increased BCFA content. It appeared that the significance of *PPARα* within the whole preparation of mammary lipid union may be related to its clear role in the direction of mitochondrial biogenesis and energy metabolism. From the results, it is indicated that yak ghee and purified BCFAs from yak ghee in the diet of mice may lead to a decrease in the number and conversion rate of mitochondria in the liver of mice, thereby reducing lipid synthesis.

Gut flora imbalance is closely related to the health and development of many diseases, such as obesity, diabetes, nonalcoholic fatty liver disease, and cancer [35]. The alpha diversity analysis of the gut flora of mice showed that there was no significant difference between the Shannon and Simpson indices in each group; that is, there were no significant differences in sample richness or uniformity. The analysis of the beta diversity of the gut flora of mice indicated that the effect of diet on the gut flora is important. Studies have shown that *Bacteroides* are beneficial to human health. It is the most abundant bacteria in the gut flora of healthy people. It can participate in carbohydrate, bile acid, and steroid metabolism, and it can also promote gut mucosal blood vessel formation. The relative abundance of *Firmicutes* in patients with dyslipidemia, obesity, hypertension, and insulin resistance is higher than that in healthy people [36]. The relative abundances of *Bacteroides* in the gut flora of the mice in the treatment group were significantly higher than those in the control group; *Firmicutes* and *Proteobacteria* were significantly decreased, and the *microbiota* also proliferated. *Proteobacteria* are gram-negative bacteria, and most of the bacteria are pathogenic bacteria such as *Hemococcus*, *Salmonella*, *Klebsiella*, and *Shigella* [37]. Most of the bacteria in *Helicobacter* are pathogenic bacteria, such as *Helicobacter pylori*, associated with the development of chronic gastritis, peptic ulcers, and gastric cancer [38]. The relative abundance of *Helicobacter* in the treatment group and the ghee group was significantly lower than that in the control group. Both *Akkermansia* and *Lactobacillus* are beneficial for health. The main strain of *Akkermansia* is *Akkermansia muciniphila*, which degrades mucin properties and makes it a key microorganism for maintaining the gut mucosal barrier. The relative abundances of *Akkermansia* and *Lactobacillus* were the highest in the treatment group. *Lactobacillus* are one of the main bacteria in the intestinal tract of humans and animals, which can regulate the balance of intestinal flora, promote the diversity of flora in the intestinal tract, and enhance the body’s immunity and resistance, which is beneficial to health [39]. This result suggested that the enrichment of BCFAs from yak ghee can regulate the gut flora by reducing the relative abundances of *Proteobacteria* and *Helicobacter* and increasing the relative abundances of *Akkermansia* and *Lactobacillus*.

KEGG is a comprehensive database integrating genomic, chemical, and systemic functional information in metabolic pathways. BCFAs can not only regulate lipid metabolism for fatty acid synthesis, elongation, and degradation but can also biologically regulate carcinoma suppression and upregulation of microRNAs in cancer and the AMPK signaling pathway, which is in accordance with Nan Xu’s investigation showing that β-patchoulene progresses lipid metabolism to lighten nonalcoholic fatty liver illness by enacting the AMPK signaling pathway. Through the intervention experiments on cancer cells, the BCFAs of yak ghee were preliminarily verified to regulate the level of cellular lipid metabolism at the in vivo gene level, which provided the data basis for the subsequent studies on lipid-lowering activity using adipocytes as the carriers.

The diet containing BCFAs purified from yak ghee has a comprehensive regulatory effect on the body that involves biological systems such as lipid metabolism, biological pathways, and intestinal flora. Our results suggest that differences in the gene expression levels of members of lipid metabolism pathways could explain the health benefits as well as probiotic enrichment. The present study provides valuable information for improving yak ghee quality.

## 4. Materials and Methods

### 4.1. The Experimental Animals and Materials

Twenty-four SPF C57BL/6J mice were purchased from JiangSu Ji Cui Yao Kang Biotechnology Co., Ltd., Nanjing, China. Pellet feed was purchased from Jiang Su Xie Tong Biological Co., Ltd., Nanjing, China. The human breast cancer cell line SK-BR-3 was acquired from Shanghai Bogu Biotechnology Co., Ltd., Shanghai, China. The powder DMEM culture medium (Gibco, Ultimo, NSW, Australia), fetal bovine serum (Gibco, Ultimo, NSW, Australia), 0.25% trypsin (EDTA Solution), PBS (phosphate-buffered saline), OMEGA DNA/RNA/Protein Isolation Kit (R6734-01) (OMEGA, Biel/Bienne, Switzerland), and inverted fluorescence microscope (DMI4000B) were provided by the cell culture laboratory at Qinghai University, Xining, China.

Yak ghee was purchased from Qinghai local herdsmen in Qinghai Province, China. BCFAs with a concentration of 16.3% were obtained by urea complexation, as detailed in Table S1. The free fatty acids from yak ghee were used as a blank control group for cellular experiments at a concentration of 3.3% BCFAs. Primer sequences were designed by Bioengineering Co., Ltd., Shanghai, China.

### 4.2. Animals and Tissue Sampling

After one week of feeding during the acclimation period, the experimental SPF mice were randomly assigned to three groups, each with eight mice: the control group, fed normal maintenance feedstuffs with the nutrient composition shown in Table S2; the ghee group, fed normal maintenance feedstuffs substituted with 10% *w*/*w* yak ghee; and the treatment group, fed normal maintenance feedstuffs substituted with 10% *w*/*w* purified yak ghee, in which BCFAs accounted for 16.3%, fed to mice at fixed times every day for 4 weeks. The experiments with the mice were approved by the ethics committee of Qinghai University (approval number SL-2021019). All experiments were performed in accordance with Chinese laws and regulations. Eyeball blood from mice was collected 12 h after the last administration of feed at the end of feeding. After standing at room temperature for 2 to 3 h, the serum was separated at 1150× *g* for 15 min in a low-temperature centrifuge, and serum samples were frozen at −80 °C. Samples of liver and feces from colon sites were collected and stored at −80 °C (liquid nitrogen freezing). Livers were collected from each group of eight for PCR experiments, and three colon fecal samples were taken from each of the three groups of mice for 16S rDNA gene sequencing.

### 4.3. Measurement of Serum Biochemical Indicators

TC, TG, LDL-C, and HDL-C were measured using kits supplied by Nanjing Jiancheng Bio-Engineering Institute Co., Ltd. (Nanjing, China) [32].

### 4.4. RNA Preparation and Quantitative Real-Time PCR

Total RNA from the liver was extracted using an RNA-solv reagent (OMEGA Bio-Tek, Norcross, GA, USA) in accordance with the manufacturer’s instructions. The quality and purity of total RNA were assessed using a NanoDrop 2000 spectrophotometer (Thermo Fisher Scientific, Shanghai, China). cDNA was synthesized from 2 μL of total RNA using a cDNA Reverse Transcription Kit (Accurate Biotechnology Co., Ltd., Danyang, China). The reaction conditions were as follows: 37 °C for 15 min, followed by 85 °C for 5 s. Quantitative real-time PCR was performed using iTaq Universal SYBR Green Supermix (Bio-Rad, Hercules, CA, USA) on a CFX 96 real-time PCR detection system (Bio-Rad). Reactions were incubated in a 96-well optical plate at 95 °C for 30 s, followed by 40 cycles of 95 °C for 5 s, 58 °C for 30 s, 95 °C for 10 s, 65 °C for 5 s, and 95 °C for 5 s. The gene expression levels were normalized to those of GAPDH, and relative mRNA expression was analyzed using the 2^−ΔΔCT^ method. The primer sequences are listed in Table S3.

### 4.5. 16S rDNA Gene Sequencing

#### 4.5.1. Total DNA of the Microbe Preparation

Total DNA from the fecal sample taken from the mouse colon was extracted utilizing the E.Z.N.A.^®^ Stool DNA Kit (Omega, Norcross, GA, USA). The procedure used followed the specifications of the instructions, and the extracted DNA was stored at −80 °C. The quality of the extracted DNA was measured by agarose gel electrophoresis, and the DNA was quantified by ultraviolet spectrophotometry.

#### 4.5.2. 16S rDNA High-Throughput Sequencing Analysis

The 16S rDNA gene variable region (V3 + V4) was subjected to PCR amplification. The bacterial 16S rDNA V3 + V4 region universal primers 338F: ACTCCTACGGGAGGCAGCAG and 806R: GGACTACHVGGGTWTCTAAT were used. The obtained PCR products were constructed into sequencing libraries and sequenced using the Illumina MiSeq platform for bioinformatics analysis of high-quality sequencing data.

### 4.6. Cell Culture

Free fatty acids from yak ghee were added to SK-BR-3 human breast cancer cells as a blank control group (total purity of BCFAs: 3.3%), and BCFAs (total purity of BCFAs: 16.3%) were added to human breast cancer cells as an experimental group. The free fatty acids of yak ghee were prepared by saponification, and the mixed fatty acids were obtained by urea complexation to purify the BCFAs. The human breast cancer cell line SK-BR-3 was passaged after full growth (80%~90%). The medium was filtered through a 0.22 μm membrane, and 15% fetal bovine serum and 1% double antibody were added at a volume ratio of 1:2 to 1:3. Culture flasks with a fuller growth of human breast cancer cells were selected for the experiment. Yak ghee-free fatty acids (total purity of BCFAs: 3.3%) were added to the full-grown culture flasks as a blank group, BCFAs (total purity of BCFAs: 16.3%) were added to the culture flasks as an experimental group, and the cells were incubated in an incubator at 37 °C with 5% CO_2_ for 24 h. The cells were then subjected to Illumina sequencing.

### 4.7. Illumina Sequencing

After sequencing, clean reads were collected by evacuating low-quality peruses and peruses with adapter/poly-N arrangements. Altogether, Q20, Q30, and the GC substance of the clean peruses were obtained, and advanced examinations were performed on the premise of clean reads. Sequencing was completed by Beijing Nuohezhiyuan Technology Co., Ltd. (Beijing, China). A KEGG pathway enrichment examination of differentially communicated genes was performed to decide which biological functions or pathways the differentially communicated genes beneath diverse conditions were related to.

### 4.8. Statistical Analysis

A one-way ANOVA was performed utilizing SPSS 22.0, and the fluctuation was analyzed by the Tamhane method. Lowercase letters indicate significance at the 0.05 level, and uppercase letters indicate significance at the 0.01 level. Different letters indicate significant or highly significant differences between groups. The Alpha diversity analysis indices of microbial communities were calculated using a taxonomic operational unit (OTU) table. Beta diversity analysis was performed by Principal Coordinate Analysis (PCoA) and Principal Component Analysis (PCA).

## 5. Conclusions

In conclusion, BCFAs purified from yak ghee could reduce body fat by inhibiting glycerophosphate metabolism, reducing lipid synthesis by thyroid hormones and carbohydrates, and increasing β-oxidation of fatty acids. Gut microbiota 16S rDNA sequencing analysis showed that BCFAs purified from yak ghee could increase the relative abundance of *Bacteroidetes*, *Verrucomicrobia*, *Akkermansia*, and *Lactobacillus*, decrease the relative abundance of *Firmicutes*, and lower the F/B ratio, which could contribute to the body’s consumption of energy and play a role in lipid metabolism regulation. The addition of BCFAs purified from yak ghee to cultured SK-BR-3 breast cancer cells altered the expression of biological metabolic pathways in SK-BR-3 breast cancer cells. The addition of BCFAs purified from yak ghee to cultured breast cancer cells SK-BR-3 altered the expression of lipid metabolic pathways in breast cancer cells SK-BR-3.

## Figures and Tables

**Table 1:** Body weight, food intake and food utilization of mice ($\bar{X}\pm S$).

Group	Initial Weight/g	Weight after Four Weeks/g	Difference/g	Total Food Intake/g	Food Utilization Rate/%
control	16.278 ± 0.373 ^bB^	19.969 ± 0.949 ^aA^	3.691 ± 0.942 ^aA^	814.507 ± 26.328 ^aA^	0.453 ± 0.361 ^aA^
ghee	16.175 ± 0.369 ^bB^	20.070 ± 0.940 ^aA^	3.895 ± 0.977 ^aA^	801.244 ± 30.614 ^aA^	0.486 ± 0.215 ^aA^
treat	16.411 ± 0.643 ^aA^	20.209 ± 1.168 ^aA^	3.798 ± 1.349 ^aA^	794.825 ± 27.145 ^aA^	0.478 ± 0.241 ^aA^

Note: Lowercase letters indicate significance at the 0.05 level, and uppercase letters indicate significance at the 0.01 level. Different letters indicate significant or highly significant differences between groups.

**Table 2:** Total cholesterol, triglyceride, LDL-cholesterol and HDL-cholesterol levels of mice ($\bar{X}\pm S$).

Group	TC (mmol/L)	TG (mmol/L)	LDL-C (mmol/L)	HDL-C (mmol/L)
control	6.646 ± 0.345 ^aA^	0.943 ± 0.009 ^bB^	1.059 ± 0.012 ^aA^	1.093 ± 0.018 ^bB^
ghee	6.844 ± 0.565 ^aA^	1.126 ± 0.078 ^aA^	1.104 ± 0.016 ^aA^	1.007 ± 0.039 ^cC^
treat	5.543 ± 0.661 ^bB^	0.837 ± 0.033 ^bB^	0.856 ± 0.044 ^bB^	1.214 ± 0.008 ^aA^

Note: Lowercase letters indicate significance at the 0.05 level, and uppercase letters indicate significance at the 0.01 level. Different letters indicate significant or highly significant differences between groups.

**Table 3:** Alpha Diversity Index Analysis ($\bar{X}\pm S$).

Group	Observed_Species	Chao1	Shannon	Simpson
control	2132.00 ± 109.42	2734.02 ± 109.52 ^bB^	7.02 ± 0.27	0.97 ± 0.01
ghee	1980.33 ± 56.57	2359.89 ± 36.29 ^aA^	6.94 ± 0.01	0.97 ± 0.01
treat	2083.67 ± 133.83	2689.39 ± 60.43 ^bB^	6.93 ± 0.26	0.96 ± 0.02

Note: Lowercase letters indicate significance at the 0.05 level, and uppercase letters indicate significance at the 0.01 level. Different letters indicate significant or highly significant differences between groups.

**Table 4:** Relative abundance of major phyla in the mouse intestinal microbiota ($\bar{X}\pm S$).

Group	*Bacteroidetes*	*Firmicutes*	F/B	*Proteobacteria*	*Actinomycetes*	*Candidatus_Saccharibacteria*	*Verrucomicrobia*
control	87.601 ± 2.047 ^cC^	11.052 ± 2.286 ^aA^	0.126	0.533 ± 0.000 ^aA^	0.218 ± 0.010 ^bB^	0.387 ± 0.237 ^aA^	0.006 ± 0.005 ^bB^
ghee	93.837 ± 0.228 ^bB^	5.530 ± 0.026 ^bB^	0.059	0.091 ± 0.011 ^cC^	0.139 ± 0.067 ^bB^	0.058 ± 0.022 ^bB^	0.001 ± 0.001 ^bB^
treat	97.015 ± 3.690 ^aA^	1.600 ± 4.777 ^cC^	0.016	0.366 ± 0.024 ^bB^	1.108 ± 1.020 ^aA^	0.086 ± 0.098 ^bB^	0.189 ± 0.182 ^aA^

Note: Lowercase letters indicate significance at the 0.05 level, and uppercase letters indicate significance at the 0.01 level. Different letters indicate significant or highly significant differences between groups.

**Table 5:** Relative abundances of the main genera in the intestinal microflora of mice ($\bar{X}\pm S$).

Group	*Helicobacter*	*Bilophila*	*Akkermansia*	*Lactobacillus*
control	0.069 ± 0.109 ^aA^	NA	0.005 ± 0.004 ^bB^	0.083 ± 0.054 ^aA^
ghee	NA	NA	0.001 ± 0.001 ^bB^	0.201 ± 0.093 ^aA^
treat	NA	0.051 ± 0.071 ^aA^	0.344 ± 0.100 ^aA^	1.381 ± 1.964 ^aA^

Note: Lowercase letters indicate significance at the 0.05 level, and uppercase letters indicate significance at the 0.01 level. Different letters indicate significant or highly significant differences between groups.

## Data Availability

The data generated from the study are clearly presented and discussed in the manuscript.

## Supplementary Materials

The following supporting information can be downloaded at: https://www.mdpi.com/article/10.3390/molecules28207222/s1, Table S1: FA composition(%, *w*/*w*) of yak butter, BCFA purified with urea (urea/FAs, adduction temperature and adduction time at 2:1, 4 °C and 8 h, respectively); Table S2: Nutrient composition and calorie content of general maintenance feeds; Table S3: Primer sequence information; Table S4: Relative abundance of the main genus of the gut microbiota in mice; Table S5: Relative abundance of the main species in the intestinal microflora in mice; Table S6: Down-regulated the significant metabolic pathway; Table S7: Up-regulated the significant metabolic pathway of the expressed gene.
