# Supplementary material for: Effects of Branched-Chain Fatty Acids Derived from Yak Ghee on Lipid Metabolism and the Gut Microbiota in Normal-Fat Diet-Fed Mice
Source: Molecules. 2023 Oct 23;28(20):7222. doi: 10.3390/molecules28207222 (PMC10609089; doi:10.3390/molecules28207222)
Supplement: Supplementary file 1 [file molecules-28-07222-s001.zip › molecules-2574640-supplementary.pdf]

Table S1. FA composition(%, w/w) of yak butter, BCFA purified with urea (urea/FAs, adduction temperature and adduction time at 2:1, 4℃ and 8h, respectively).

FA	Yak butter	BCFA purified Yak butter
C6:0	NA	2.9 ± 0.1
C8:0	0.3 ± 0.0	9.3 ± 0.1
C10:0	1.8 ± 0.1	12.1 ±0.3
C12:0	4.2 ± 0.3	10.4 ± 0.6
C13:0	0.1 ± 0.0	0.01 ± 0.0
Iso-C14:0	0.2 ± 0.0	2.1 ± 0.8
C14:1	0.2 ± 0.0	3.7 ± 0.1
C14:0	9.4 ± 0.7	2.0 ± 1.2
Iso-C15:0	0.5 ± 0.0	0.05 ± 0.0
Anteiso-C15:0	1.0 ± 0.1	10.9 ± 0.5
C15:0	1.7 ± 0.1	0.05 ± 0.0
Iso-C16:0	0.4 ± 0.0	0.3 ± 0.0
C16:1n9	1.3 ± 0.0	5.6 ± 0.0
C16:0	25.6 ± 1.2	2.0 ± 0.0
Iso-C17:0	0.6 ± 0.0	2.9 ± 0.0
Anteiso-C17:0	0.7 ± 0.0	0.02 ± 0.0
C17:1n7	0.4 ± 0.0	0.1 ± 0.0
C17:0	1.1 ± 0.0	0.1 ± 0.0
C18:2	0.1 ± 0.0	23.3 ± 0.3
C18:1n9	29.1 ± 1.9	9.5 ± 0.1
C18:0	17.1 ± 1.0	0.1 ± 0.0
Cis9,trans11CLA	1.5 ± 0.0	1.5 ± 0.2
C19:1	0.2 ± 0.0	NA
C19:0	0.2 ± 0.0	NA
C20:1n9	0.4 ± 0.0	NA
C20:0	0.7 ± 0.0	NA
C21:0	0.1 ± 0.0	NA
C22:6n3	NA	0.1 ± 0.0
C22:5n3	0.1 ± 0.0	1.2 ± 0.0
C22:1n9	0.1 ± 0.0	NA
C22:0	0.3 ± 0.0	NA
C23:0	0.1 ± 0.0	NA
C24:0	0.1 ± 0.0	NA
Total BCFA	3.3 ± 0.2	16.3 ± 0.9

Table S2. Nutrient composition and calorie content of general maintenance feeds

**Nutritional indicators**		**minerals**		**amino acids**		**vitamins**	
Moisture,g/kg	≤100	Mg,g/kg	≥2.0	Lys,g/kg	≥8.2	vitA,IU/kg	≥7000
crude protein,g/kg	≥180	K,g/kg	≥5.0	Met+cys,g/kg	≥5.3	vitD,IU/kg	≥800
crude fat,g/kg	≥40	Na,g/kg	≥2.0	Arg,g/kg	≥9.9	vitE,IU/kg	≥60
crude fiber,g/kg	≤50	Fe,mg/kg	≥100	His,g/kg	≥4.0	vitK,mg/kg	≥3.0
crude ash,g/kg	≤80	Mn,mg/kg	≥75	Trp,g/kg	≥1.9	vitB1,mg/kg	≥8
Ca,g/kg	10~18	Cu,mg/kg	≥10	Phe+Tyr,g/kg	≥11.0	vitB2,mg/kg	≥10
total phosphorus, g/kg	6~12	Zn,mg/kg	≥30	Thr,g/kg	≥6.5	vitB6,mg/kg	≥6
Ca:P	1.2:1~1.7:1	I,mg/kg	≥0.5	Leu,g/kg	≥14.4	vitB12,mg/kg	≥0.020
		Se,mg/kg	0.1~0.2	Ile,g/kg	≥7.0	niacin,mg/kg	≥45
				Val,g/kg	≥8.4	Pantothenic acid,mg/kg	≥17
						VBc ,mg/kg	≥4.0
						Biotin,mg/kg	≥0.10
						Choline,mg/kg	≥1250
						vitC,mg/kg	/

	**energy supply ratio**
Protein	20.6%
Fats	12.0%
Carbohydrates	67.4%
Total calories	3530Kcal/kg

Table S3. Primer sequence information.

Primer name	Primer sequences information (5’-3’)
DGAT-1-F	GTTTCCGTCCAGGGTGGTAG
DGAT-1-R	GACGATGGCACCTCAGATCC
ACC-α-F	ACGTGAATGCTTGACCAGGG
ACC-α-R	AGGCAAAGCCTCCAGTAAGC
FAS-F	GTGAGTCTATCCTGCGCTCC
FAS-R	GTCGATGAGGGCAATCTGGA
FADS1-F	CTCGTGATCGACCGGAAGG
FADS1-R	ATGCCACAAAAGGATCCGTGG
FADS2-F	AGCCCCTTGAGTATGGCAAG
FADS2-R	ATAGTAGCTGATGGCCCAAGC
ACSS1-F	CGGTTGGATCACAGGACACA
ACSS1-R	GTCTCCCAGTAACGACCAGC
ACSL1-F	GTAGGACTCGGCATGTGACAA
ACSL1-R	GCAGAATTCATCTGTGCCATC
GPAT4-F	CTGGTGGCTAAGAGGCTGAC
GPAT4-R	AGAAGGCGTCACCAAACTGA
GPAM-F	AGCTTCTAAGTCACCCACACC
GPAM-R	TTGCCAGGCTCCTGATAAATAG
THRSP-F	TCTGAAGATCGCTTTACACGAGA
THRSP-R	GATGCACTCAGAGGGAGACG
HMGCR-F	AGAGAACAAGGGTTCACGCC
HMGCR-R	CCTTGGATCCCACGCGGA
SCD1-F	CTGAACACCCATCCCGAGAG
SCD1-R	AACTGGAGATCTCTTGGAGCA
PPARα-F	TCGCAGCTGTTTTGGGGG
*PPAR*α-R	CAACTTGGCTCTCCTCTAAGTTC
*CPT*1-F	TCCGCTCGCTCATTCCGC
*CPT*1-R	TGCCATTCTTGAATCGGATGAACT
*FABP*1-F	TGAAGGCAATAGGTCTGCCC
FABP1-R	GTCATGGTCTCCAGTTCGCA

A-FABP-F	GTGGGATGGAAAGTCGACCA
A-FABP-R	CATAACACATTCCACCACCA GC

Table S4. Relative abundance of the main genus of the gut microbiota in mice.

group	Control	Ghee	Treat(BCFA)
Up-regulation	*Osillibacter*	*Tidjanibacter*	*Acetvibrio*
	Desufovibrio	*Lactobacilus*	*Akkermansia*
	Porphyromonadaceae	*Parabateroides*	*Baderoidetes*
	Bacteroides	*Alloprevotella*	*Clostridium_XVa*
	Ruminoccoccaceae	*Bamesiella*	*Lachnosplraceae*
		Anaerotaenia	*Tidjanibacter*
		Baceroidales	*Lactobacilus*
		Prevotella	*Parabateroides*
		Clostridium_XVb	*Alloprevotella*
		Lachnoclostidium	*Bamesiella*
		Osillibacter	*Anaerotaenia*
			Baceroidales
			Prevotella
Down-regulation	*Acetvibrio*	*Acetvibrio*	*Clostridium_XVb*
	Baderoidetes	*Baderoidetes*	*Alistipes*
	Clostridium_XVa	*Clostridium_XVa*	*Lachnoclostidium*
	Lachnosplraceae	*Desufovibrio*	*Osillibacter*
	Tidjanibacter	*Porphyromonadaceae*	*Desufovibrio*
	Lactobacilus	*Bacteroides*	*Porphyromonadaceae*
	Parabateroides	*Ruminoccoccaceae*	*Bacteroides*
	Alloprevotella		*Ruminoccoccaceae*
	Bamesiella		
	Anaerotaenia		
	Baceroidales		
	Prevotella		
	Clostidium_XVb		
	Alistipes		

Table S5. Relative abundance of the main species in the intestinal microflora in mice.

group	*Lactobacillus sp. L-YJ*	*Lactobacillus vaginalis*
control	0.015±0.008[b]	0.000±0.000[b]
ghee	0.268±0.113[a]	0.014±0.023[ab]
treat	0.987±1.417[a]	0.223±0.332[a]

Table S6. Down-regulated the significant metabolic pathway.

Pathway	Database	Id	Qnum	Bnum	*P*-Value
Oxidative phosphorylation	KEGG PATHWAY	hsa00190	62	116	1.41E-09
DNA replication	KEGG PATHWAY	hsa03030	26	34	2.84E-09
Parkinson's disease	KEGG PATHWAY	hsa05012	63	122	6.35E-09
Ribosome	KEGG PATHWAY	hsa03010	61	126	2.34E-07
Alzheimer's disease	KEGG PATHWAY	hsa05010	70	151	2.60E-07
Cell cycle	KEGG PATHWAY	hsa04110	57	120	1.28E-06
Huntington's disease	KEGG PATHWAY	hsa05016	75	171	1.47E-06
Citrate cycle (TCA cycle)	KEGG PATHWAY	hsa00020	19	29	1.76E-05
Pyrimidine metabolism	KEGG PATHWAY	hsa00240	43	93	5.80E-05
Carbon metabolism	KEGG PATHWAY	hsa01200	48	108	7.65E-05
**Fatty acid degradation**	KEGG PATHWAY	hsa00071	21	37	0.000138334
Lysosome	KEGG PATHWAY	hsa04142	48	114	0.00037155
Base excision repair	KEGG PATHWAY	hsa03410	18	32	0.000483207
Non-alcoholic fatty liver disease (NAFLD)	KEGG PATHWAY	hsa04932	54	133	0.000488679
Valine, leucine and isoleucine degradation	KEGG PATHWAY	hsa00280	23	45	0.000536461
**Fatty acid metabolism**	KEGG PATHWAY	hsa01212	22	44	0.001035275
Pyruvate metabolism	KEGG PATHWAY	hsa00620	19	37	0.001504183
Tryptophan metabolism	KEGG PATHWAY	hsa00380	17	32	0.001636736

Table S7. Up-regulated the significant metabolic pathway of the expressed gene.

Pathway	Database	Id	Qnum	Bnum	*P*-Value
Protein processing in endoplasmic reticulum	KEGG PATHWAY	hsa04141	54	147	2.92E-10
FoxO signaling pathway	KEGG PATHWAY	hsa04068	39	115	1.02E-06
TGF-beta signaling pathway	KEGG PATHWAY	hsa04350	26	73	2.46E-05
Ubiquitin mediated proteolysis	KEGG PATHWAY	hsa04120	39	130	2.83E-05
Autophagy - animal	KEGG PATHWAY	hsa04140	37	122	3.48E-05
Endocytosis	KEGG PATHWAY	hsa04144	56	219	9.78E-05
Signaling pathways regulating pluripotency of stem cells	KEGG PATHWAY	hsa04550	34	114	0.000103979
Long-term potentiation	KEGG PATHWAY	hsa04720	20	54	0.000113333
Renal cell carcinoma	KEGG PATHWAY	hsa05211	22	63	0.000143956
Ferroptosis	KEGG PATHWAY	hsa04216	15	37	0.000256599
Proteoglycans in cancer	KEGG PATHWAY	hsa05205	44	168	0.000307667
Pathways in cancer	KEGG PATHWAY	hsa05200	95	437	0.000394038
Wnt signaling pathway	KEGG PATHWAY	hsa04310	33	117	0.000413751
Viral carcinogenesis	KEGG PATHWAY	hsa05203	44	173	0.000610823
Cellular senescence	KEGG PATHWAY	hsa04218	37	143	0.001157881
Phospholipase D signaling pathway	KEGG PATHWAY	hsa04072	32	120	0.001432184
Mitophagy - animal	KEGG PATHWAY	hsa04137	19	61	0.001956053
mTOR signaling pathway	KEGG PATHWAY	hsa04150	35	137	0.001973093
MicroRNAs in cancer	KEGG PATHWAY	hsa05206	37	147	0.0019777
AMPK signaling pathway	KEGG PATHWAY	hsa04152	28	106	0.003218418
